# Genome Sequence and Methylation Patterns of *Halorubrum* sp. Strain BOL3-1, the First Haloarchaeon Isolated and Cultured from Salar de Uyuni, Bolivia

**DOI:** 10.1128/MRA.00386-19

**Published:** 2019-05-09

**Authors:** Priya DasSarma, Brian P. Anton, Satyajit DasSarma, Victoria J. Laye, Daniel Guzman, Richard J. Roberts, Shiladitya DasSarma

**Affiliations:** a Department of Microbiology and Immunology, Institute of Marine and Environmental Technology, University of Maryland School of Medicine, Baltimore, Maryland, USA; b New England Biolabs, Ipswich, Massachusetts, USA; c Centro de Biotecnología, Faculty of Sciences and Technology, Universidad Mayor de San Simón, Cochabamba, Bolivia; Portland State University

## Abstract

*Halorubrum* sp. strain BOL3-1 was isolated from Salar de Uyuni, Bolivia, and sequenced using single-molecule real-time sequencing. Its 3.7-Mbp genome was analyzed for gene content and methylation patterns and incorporated into the Haloarchaeal Genomes Database (http://halo.umbc.edu). The polyextremophilic character and high-elevation environment make the microbe of interest for astrobiology.

## Abstract (precis)

*Halorubrum* sp. strain BOL3-1 was isolated from Salar de Uyuni, Bolivia, and sequenced using single-molecule real-time sequencing. Its 3.7-Mbp genome was analyzed for gene content and methylation patterns and incorporated into the Haloarchaeal Genomes Database (http://halo.umbc.edu).

## ANNOUNCEMENT

*Halorubrum* sp. strain BOL3-1 is the first haloarchaeon from Bolivia to be cultured and sequenced. It was isolated from salt samples from Salar de Uyuni, Department of Potosí, Bolivia, the largest salt flat in the world and an environment remarkable for its high elevation and high albedo and UV radiation exposure (1). The environment is unique and of significant interest to the astrobiology community due to its multiple extremes (2).

Stratified salt crust was sampled from the Salar in March 2015 at a remote site, (20°33′28.58ʺS and 67°12′29.56ʺW [–20.5579389°, –067.2082111°]), 3,647 m above sea level. Typical conditions are pH 7.3 to 7.6, ≥28% NaCl (wt/vol) concentration, and temperatures of –15 to 22°C. Salt samples were dissolved in CM^+^ medium (3), and growth was stimulated under illumination at 37°C with shaking at 220 rpm (Innova 4230 refrigerated incubator shaker; New Brunswick, NJ, USA). The enrichment culture was plated on CM^+^ agar plates and purified by 3 rounds of streaking. The isolated strain, BOL3-1, formed biofilms in liquid culture, and colonies were bright red and translucent.

Nucleic acids were extracted using standard methods (3), and sequencing was performed using the PacBio RS II platform. A SMRTbell sequencing library was prepared from 3 μg genomic DNA randomly sheared to 20 kb with a Megaruptor instrument (Diagenode, Denville, NJ). The library was sequenced using a single-molecule real-time (SMRT) cell with C4-P6 chemistry and a 360-min collection time. Sequencing reads were filtered (quality, ≥0.80; length, ≥100 bp) and assembled *de novo* (98,158 reads with a mean subread length of 3,908 bp) using RS_HGAP_Assembly.3 (4) in the SMRT Analysis 2.3.0 environment (minimum seed read length, 5,000 bp; minimum coverage for correction, 8×). Error correction and closure were performed using RS_BridgeMapper.1, and methylation patterns were determined using RS_Modification_and_Motif_Analysis.1 within SMRT Analysis using default settings (minimum modification quality value [QV], 30).

Genome annotation was performed in-house using EMBOSS version 6.6.0.0 (5), GeneMark.hmm version 2 (6), and tRNAscan version 1.3 (7), as well as the NCBI Prokaryotic Genome Annotation Pipeline (PGAP) build 3190 (8). The annotated genome sequence was analyzed on the Haloarchaeal Genomes Database (HaloWeb version r1555192846) (9). The 3,668,425-bp genome is GC rich (65.9%) and consists of a large circular chromosome (66.8% GC content) and 3 plasmids, p163 (55.1% GC content), p117 (54.8% GC content), and p13 (67.6% GC content) based on computer assembly. Three complete rRNA operons (two on the chromosome and one on p117) and 57 tRNA genes are present. The closest relatives based on 16S rRNA similarity (≤97% identity) are Halorubrum ezzemoulense and Halorubrum chaoviator (10, 11).

Genome annotation predicted 3,266 encoded proteins with a calculated mean isoelectric point (pI) value of 4.58, a highly acidic proteome characteristic of haloarchaea (12). The genome contains the great majority of conserved haloarchaeal groups (HOGs), including 775 core (cHOGs) and 77 signature (ucHOGs) groups (12, 13). Expanded gene families common in haloarchaea include 9 origin recognition complex (Orc/Cdc6) proteins, 4 TATA-binding and 7 TFB proteins, and 5 photolyase/cryptochrome family proteins (14). Genes encoding retinal proteins, including bacteriorhodopsin, halorhodopsin, and sensory rhodopsin 2, were found. Bacteriorhodopsin can be observed spectroscopically and constitutes a remotely detectable biosignature (15). A catabolic gene cluster is present, with a GH-42 β-galactosidase likely responsible for *o*-nitrophenyl-β-d-galactopyranoside (ONPG)-hydrolytic activity (16–18).

Over 100 transposase genes are present, suggesting a large number of insertion sequences in the genome. There are 2 clustered regularly interspaced short palindromic repeat (CRISPR) arrays (a type I-B CRISPR-associated protein, Cas5, on p163 and a type I-B CRISPR-associated protein, Cas7/Csh2, on p117). The methylated DNA motifs and the methyltransferases (MTases) predicted to be responsible for some of these proteins are shown in Table 1.

**TABLE 1:** Methylated motifs in *Halorubrum* species strain BOL3-1 detectable by SMRT sequencing

Motif^*a*^	Type	% sites methylated	Putative MTase responsible
GGAG(N5)TTC	m6A	100	EKH57_07125 (M.HspBol31I)
GATC	m6A	100	EKH57_00995 (M.HspBol31II)
GTCAGT	m6A	100	Undetermined
CAGAGC	m6A	100	Undetermined
GCGGAGG	m6A	99	Undetermined
CATTC	m6A	98	Undetermined

a Locations of methylated bases on either the top or bottom strands are underlined.

### Data availability.

The *Halorubrum* sp. strain BOL3-1 genome sequence has been deposited in GenBank with the accession numbers CP034692, CP034691, CP034690, and CP034693 and is also available on HaloWeb (https://halo.umbc.edu/cgi-bin/haloweb/haloweb.pl). The raw data are available in the NCBI Sequence Read Archive with the accession number SRP175004.

## References

[1] Banks D, Markland H, Smith PV, Mendez C, Rodriguez J, Huerta A, Sæther OM 2004 Distribution, salinity and pH dependence of elements in surface waters of the catchment areas of the Salars of Coipasa and Uyuni, Bolivian Altiplano. J Geochem Explor 84:141–166. doi:10.1016/j.gexplo.2004.07.001.

[2] Reid IN, Sparks WB, Lubow S, McGrath M, Livio M, Valenti J, Sowers KR, Shukla HD, MacAuley S, Miller T, Suvanasuthi R, Belas R, Colman A, Robb FT, DasSarma P, Müller JA, Coker JA, Cavicchioli R, Chen F, DasSarma S 2006 Terrestrial models for extraterrestrial life: methanogens and halophiles at Martian temperatures. Int J Astrobiol 5:89–97. doi:10.1017/S1473550406002916.

[3] Ng WL, Yang CF, Halladay JT, Arora P, DasSarma S 1995 Isolation of genomic and plasmid DNAs from *Halobacterium halobium*, p 179–184. *In* DasSarma S, Fleischmann EM (ed), Archaea, a laboratory manual: halophiles. Cold Spring Harbor Laboratory Press, Cold Spring Harbor, NY.

[4] Chin CS, Alexander DH, Marks P, Klammer AA, Drake J, Heiner C, Clum A, Copeland A, Huddleston J, Eichler EE, Turner SW, Korlach J 2013 Nonhybrid, finished microbial genome assemblies from long-read SMRT sequencing data. Nat Methods 10:563–569. doi:10.1038/nmeth.2474. 23644548

[5] EMBOSS Explorer. http://www.bioinformatics.nl/cgi-bin/emboss/. Accessed 21 February 2019.

[6] Besemer J, Borodovsky M 2005 GeneMark: Web software for gene finding in prokaryotes, eukaryotes and viruses. Nucleic Acids Res 33:W451–W454. doi:10.1093/nar/gki487. 15980510 PMC1160247

[7] Lowe TM, Chan PP 2016 tRNAscan-SE on-line: integrating search and context for analysis of transfer RNA genes. Nucleic Acids Res 44:W54–W57. doi:10.1093/nar/gkw413. 27174935 PMC4987944

[8] Tatusova T, DiCuccio M, Badretdin A, Chetvernin V, Nawrocki EP, Zaslavsky L, Lomsadze A, Pruitt KD, Borodovsky M, Ostell J 2016 NCBI Prokaryotic Genome Annotation Pipeline. Nucleic Acids Res 44:6614–6624. doi:10.1093/nar/gkw569. 27342282 PMC5001611

[9] DasSarma SL, Capes MD, DasSarma P, DasSarma S 2010 HaloWeb: the haloarchaeal genomes database. Saline Systems 6:12. doi:10.1186/1746-1448-6-12. 21192823 PMC3023673

[10] Kharroub K, Quesada T, Ferrer R, Fuentes S, Aguilera M, Boulahrouf A, Ramos-Cormenzana A, Monteoliva-Sánchez M 2006 *Halorubrum ezzemoulense* sp. nov., a halophilic archaeon isolated from Ezzemoul sabkha, Algeria. Int J Syst Evol Microbiol 56:1583–1588. doi:10.1099/ijs.0.64272-0. 16825633

[11] Mancinelli RL, Landheim R, Sánchez-Porro C, Dornmayr-Pfaffenhuemer M, Gruber C, Legat A, Ventosa A, Radax C, Ihara K, White MR, Stan-Lotter H 2009 *Halorubrum chaoviator* sp. nov., a haloarchaeon isolated from sea salt in Baja California, Mexico, Western Australia and Naxos, Greece. Int J Syst Evol Microbiol 59:1908–1913. doi:10.1099/ijs.0.000463-0. 19567575 PMC3182535

[12] DasSarma S, DasSarma P 2015 Halophiles and their enzymes: negativity put to good use. Curr Opin Microbiol 25:120–126. doi:10.1016/j.mib.2015.05.009. 26066288 PMC4729366

[13] Capes MD, DasSarma P, DasSarma S 2012 The core and unique proteins of haloarchaea. BMC Genomics 13:39. doi:10.1186/1471-2164-13-39. 22272718 PMC3287961

[14] Capes MD, Coker JA, Gessler R, Grinblat-Huse V, DasSarma SL, Jacob CG, Kim J-M, DasSarma P, DasSarma S 2011 The information transfer system of halophilic archaea. Plasmid 65:77–101. doi:10.1016/j.plasmid.2010.11.005. 21094181

[15] DasSarma S, Schwieterman EW 2018 Early evolution of purple retinal pigments on Earth and implications for exoplanet biosignatures. Int J Astrobiol, in press. doi:10.1017/S1473550418000423.

[16] Karan R, Capes MD, DasSarma P, DasSarma S 2013 Cloning, overexpression, purification, and characterization of a polyextremophilic β-galactosidase from the Antarctic haloarchaeon *Halorubrum lacusprofundi*. BMC Biotechnol 13:3. doi:10.1186/1472-6750-13-3. 23320757 PMC3556326

[17] Laye VJ, Karan R, Kim JM, Pecher WT, DasSarma P, DasSarma S 2017 Key amino acid residues conferring enhanced enzyme activity at cold temperatures in an Antarctic polyextremophilic β-galactosidase. Proc Natl Acad Sci U S A 114:12530–12535. doi:10.1073/pnas.1711542114. 29109294 PMC5703305

[18] Laye VJ, DasSarma S 2018 An Antarctic extreme halophile and its polyextremophilic enzyme: effects of perchlorate salts. Astrobiology 18:412–418. doi:10.1089/ast.2017.1766. 29189043 PMC5910040

